# An Intelligent System for Proper Management and Disposal of Unused and Expired Medications

**DOI:** 10.3390/ijerph19052875

**Published:** 2022-03-01

**Authors:** Haneen Banjar, Rahaf Alrowithi, Sara Alhadrami, Esraa Magrabi, Reema Munshi, Mayda Alrige

**Affiliations:** 1 Computer Science Department, Faculty of Computing and Information Technology, King Abdulaziz University, P.O. Box 80221, Jeddah 21589, Saudi Arabia; ralrowithi0002@stu.kau.edu.sa (R.A.); salhadrami0006@stu.kau.edu.sa (S.A.); emaghrbi@stu.kau.edu.sa (E.M.); 2 Centre of Artificial intelligence in Precision Medicines, King Abdulaziz University, Jeddah 21589, Saudi Arabia; 3 Clinical Pharmacy Department, College of Pharmacy, Umm al-Qura University, Makkah 21961, Saudi Arabia; remrem.munshi@gmail.com; 4 Information System Department, Faculty of Computing and Information Technology, King Abdulaziz University, Jeddah 21589, Saudi Arabia; malraegi@kau.edu.sa

**Keywords:** disposal of medication, web-based expert system, chatbot, image classification, donation device, take-back medication program

## Abstract

For years, several countries have been concerned about how to dispose of unused pharmaceuticals that can endanger human health and the environment. Moreover, some people are in desperate need of medical attention and medications, but they lack the financial resources to obtain them. In Saudi Arabia, there are no take-back medicine programs, and there is no published research on how medications properly are disposed. The aim of this research is to use the power of artificial intelligence to assist in the proper management and disposal of expired and unused medications and to develop a prototype device for collecting medication by automatically classifying medications for proper disposal and donation. In this research, artificial intelligence technologies such as web-based expert systems, image recognition and classification algorithms, chatbots, and the internet of things are used to assist in a take-back medications program. In conclusion, the prototype design of a web-based expert system and the device reduced improper disposal risks by providing significant advice on the safe disposal of unwanted pharmaceuticals. By using an organized method of collecting expired medications, the benefits were made possible.

## 1. Introduction

Medication wastage can be reduced, resulting in more cost-effective spending, which the Ministry of Health wants to achieve in line with Saudi Arabia’s 2030 economic reform program [1]. The United States Food and Drug Administration (FDA) updated the medicines’ disposal instructions. The “misuse” of prescription drugs is one of the behaviors that are on the rise in the United States [2]. Consequently, there is considerable pharmaceutical waste but there are no accurate and current statistics available. The waste of these drugs has caused biological and ethical problems that should be understood from different perspectives. Alnahas et al. [3] demonstrated that the German Federal Ministry for the Environment has made it clear that pharmaceutical organizations in both the industrialized and developing world offer a variety of synthetic chemicals at a rate of 100,000 tons per year. From this, a small fraction is used while a larger fraction will be unused and/or expired resulting in a large value of pharmaceutical waste. The authors also reported a cross-sectional study in 2001 that estimated more than 1 billion unused drugs produced each year by the American population alone. Moreover, the presence of small amounts of active pharmaceutical ingredients in the soil and waterways could impact the environment. These small amounts of effective drugs enter the food chain, where they may develop again and thus unintentionally return to humans who recently discarded them.

Medication loss is important, as many developing countries have severe shortages of drugs such as antibiotics. In this way, it would be wise to divert expired and unused medicines to the developing world, consequently maintaining a strategic distance from waste on the one hand and shortages on the other. It has also become extremely important to raise awareness of how to correctly dispose of expired medicines and what can be done with unused medicines.

In this research, three areas have been reviewed: medical, technical, and related applications.

Firstly, several studies in medical reviews have discussed the issue of improper management of medication disposal. For example, Abuassonon et al. [4] analyzed practices of residents of Jeddah concerning the disposal of unused or expired medications and their views on the take-back centers for drugs. The analysis of this study showed that a low percentage of residents of Jeddah know how to properly dispose of medications in the correct way. Wajid et al. [5] also focused on determining the prevalence and practice of unused and expired drugs among Saudi adults. The study was conducted over a period of four months among residents of the Riyadh region in the Kingdom of Saudi Arabia. Through this study, it became clear that there was a large spread of unused medicines among the community and that the practice of disposing of them was moderate in Saudi society. Therefore, the Ministry of Health should collaborate in a national initiative to educate people about acceptable disposal methods, which can lead us to use technology to help in residents’ education and protect the environment.

Secondly, the technical review covered the expert system, chatbot, and classification and the benefits of using these technologies in pharmacies. Sagdoldanova and Atymtayeva [6] discussed a system for storing and retrieving medication data using a website that allows searching for medicines by price or type. The idea of using the expert system is that the user (pharmacist) selects one disease from the list, then the system analyses all the drug recommendations and selects one suitable drug. The system shows other drugs that have the same number of symptoms and contraindications (sub drugs) as basic drugs. It has also a user interface for drug consumers, where they do not need to be pharmacists or have knowledge about medication. In addition, the expert system has an inference engine which is a mechanism that can manage the expert systems and decide if the problem has reached an acceptable solution or not. A knowledge base can also be used to obtain the guideline for proper management of disposing of medications. Researchers also found how useful chatbots are in the clinical and health field and there are several similar chatbot systems that served in the health field. Comendador et al. [7] developed a pharmabot, which is a pediatric generic medicine consultant chatbot. It gives the appropriate medication to pediatric patients after a series of conversations with the system. The chatbot prescribes generic medicines including the proper intake, dosage, drug reaction, precaution, and an indication of medicines. Moreover, classification using artificial intelligence techniques has been used on drug recovery processes to see if harmful drugs can be distinguished from harmless drugs by visual features only. In the United States, there are several improper ways to dispose of excess medicines such as storing them in medicine cabinets, deposing them in the toilet, or putting them in the regular garbage. These methods can negatively affect the environment and public health [8]. Therefore, a drug retrieval program has been established, which is sponsored by specific bodies such as the Narcotics Control Department. Almost every police station and pharmacy in the country has a drug recovery device that uses artificial intelligence techniques to classify medicines.

Finally, a number of software programs have been developed to manage medicine disposal; for example, the Preservation of Grace Association [9]. One of the aims of the Grace Preservation Society is to collect and reuse medication. Although this project achieves great benefits and helps the community, it takes time and effort and requires a lot of volunteers to work faster. The technology was not used by the association to facilitate work and achieve its benefits. The other software is “Medicine Charity Association” [10]. Many services are provided on the “Dwaa” website, including providing medication to the needy, by purchasing medicines from donated amounts and delivering them to the needy. The Saudi Food and Drugs Authority website has instructions for medicine disposal to reduce damage and environmental pollution [11]. In addition, the “Tameeni” application can be used to search for registered drugs and their details such as the method of use and storage legal status (prescription medications or non-prescription medications). The Saudi Food and Drugs Authority also provides a chatbot with a WhatsApp application named “the smart help Sara”. This chatbot can help the patient to know the price of a medicine or give an alternative medicine. However, the applications do not connect with smart devices or automated classification.

Most households in all societies are overcrowded with medicines in cupboards and fridges; some are expired, and others are usable but not used or needed. Depending on this overcrowding, most people dispose of medication incorrectly. Some of them pour liquid medicines into bathtubs or toilets, causing pollution to the environment and the interaction of chemicals with the environment, this could result in new diseases that negatively affect society. In addition, most people dispose of medicines by throwing them in garbage containers, which increases the risk of chemicals when exposed to the sun or harming feeding animals. In economic terms, this waste of medicines results in a massive loss of financial resources that the state expends to provide treatment. In Saudi Arabia, there are no take-back medicine programs, and there is no published research on how medications are disposed of or wasted. On the other hand, some people need treatment and medicine but do not have enough money to buy them or cannot obtain them. For these reasons, a technical solution would help bridge all these problems and address them through the implementation of a website and device.

The aim of this research is to use the power of artificial intelligence to assist in the proper management and disposal of expired and unused medications and to develop a prototype device for collecting medications by automatically classifying medications for proper disposal and donation. To achieve this aim, there are four sub-objectives: (i) collect medication information to create a knowledge base, (ii) develop a web-based expert system to educate consumers about proper medication disposal practices, (iii) develop a chatbot to interact with consumers, and (iv) develop a prototype device for collecting medication by automatically classifying medications for proper disposal and donation.

## 2. Materials and Methods

There are two appropriate options for the safe disposal of medicines. The best way to dispose of old, unused, or unwanted medicines is through what is known as a take-back medicine program, which is a system developed by developed countries that allows disposing of medicines by taking them to points designated for drug retrieval, such as pharmacies or centers dedicated to this and destroying them [12]. The second option is to dispose of medication at home. If you cannot reach one of these disposal points, you can follow some simple steps to dispose of most medicines at home. However, consumers should be aware of these steps. Therefore, this project proposes a solution to protect the environment and reduce this phenomenon, and the proposed solution system is divided into two parts (software and hardware). Firstly, the software includes a web-based expert system (knowledge base and inference engine), information management system (IMS) (database), chatbot, and classification model. Secondly, the hardware device is integrated with the first part. The classification model controls the hardware device to automatically open an accurate place for disposing of the medication based on the medication details.

### 2.1. Web-Based Expert System

In artificial intelligence, expert systems are computer systems developed to emulate the decision-making ability of a human expert and solve complex problems in a particular domain. An expert system with web development technology can generate web-based expert systems [13]. An expert system that is web-based requires an awareness of the fundamental concepts of system development and web engineering principles. The following framework is the result of combining the expertise found in these two fields. Firstly, knowledge engineers begin collecting knowledge to fulfill the needs of the web-based expert system in terms of medication disposal and donation. A knowledge base is constructed after knowledge acquisition, representation, and verification. In this study, the knowledge is collected from documentation published by the Saudi Food and Drug Authority and World Health Organization.

Pharmacists are medication professionals responsible for the preparation of different forms of medication, such as tablets, pills, capsules, and sterile solutions for injection. They use their extensive knowledge of drugs to help patients in the healing process. Medicines refer to any product that contains one or more substances for use in the treatment and prevention of human diseases [14]. Medications sold in pharmacies can be divided into two categories: prescription medications: that require a prescription to be sold; and nonprescription over-the-counter (OTC) medications: that do not require a prescription from a doctor. Whether the medicine is by prescription or non-prescription, the wrong method of disposal has significant side effects on the community and environment. Many medications come in several different forms. The most common forms of medications used are demonstrated in Table 1.

There are two main documents that were used in this study. The Saudi Food and Drug Authority [15] is a public document that discusses policy and instructions for donation. This document also contains important information about the donation process (see Supplementary Section S1). The document demonstrates those who can donate medications such as a manufacturer or supplier of drugs, governmental entities, private pharmacies, private hospitals, individuals (the donor, by purchasing medicines from their main source and the patient or a member of their family), and charities by purchasing medicines from their main source.

The second main document is the Guidelines for Safe Disposal of Unwanted Pharmaceuticals in and after Emergencies, published by the World Health Organization (WHO) [16]. This document contains the recommendations of disposal methods under sorted categories that require various disposal methods. The two categories are: (1) pharmaceuticals and other materials which can still be used and (2) expired or unwanted pharmaceuticals. Firstly, pharmaceuticals within their expiry date and that are deemed to be of use should be segregated and immediately used by the organization or reallocated according to the needs and instructions of the regional health authorities. Secondly, pharmaceuticals should never be used and should always be considered as pharmaceutical waste including all expired pharmaceuticals, all bulk or lost tablets, and capsules. If unexpired, these should only be used when the container is still sealed, properly labeled, or still within the original unbroken blister packs and all unsealed tubes of creams, ointments.

**Figure 1:**
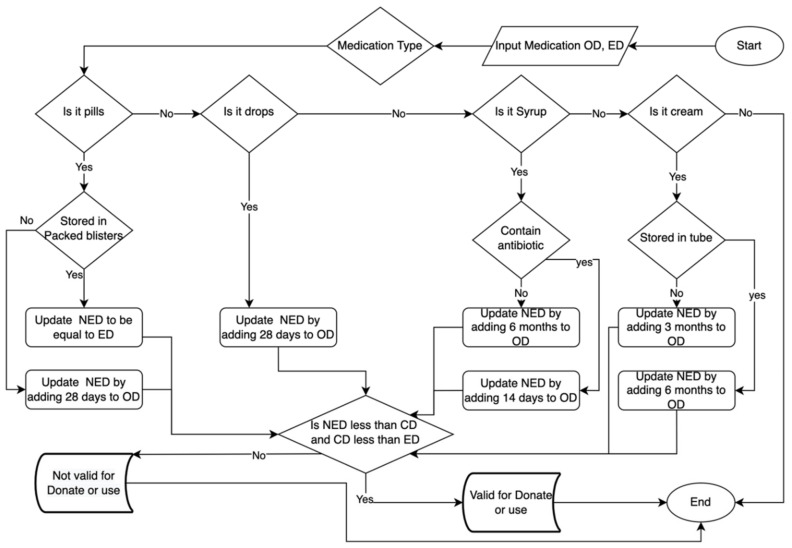
The knowledge representation. Open date (OP), current date (CD), expired date (ED), and calculated new expired date (NED).

Knowledge representation is a method of storing and conveying information that a computer can use to solve issues, such as conversing with humans in natural language. What makes knowledge representation different from database storage is that it allows machines to learn from that knowledge and act intelligently, like a person. Figure 1 shows the knowledge representation for the disposal and donation of medication.

Then, it is necessary to have expert field verification before bringing the knowledge collected during knowledge acquisition to the expert system design step, which uses flowcharts to model that information. Any disagreements in opinion were resolved by a majority vote among three experts from the College of Pharmacy, Umm al-Qura University.

Secondly, expert system development is included in the design phase (knowledge base, inference engine, and user interfaces). After presenting the knowledge in the flowchart, the flowchart is converted into if-then rules. Then, the inference engine, which is the main processing component of the expert system, gains knowledge from the knowledge base and engages with it to come up with a specific disposal recommendation. For example, the user enters the medication open date. The inference will fire the rules that match the medication types and calculate the expired date as suggested by the manufacturer. On the due date, the medication disposal recommendation appears on the website to notify the user about the medication and the proper methods for disposal.

Thirdly, adaptations made during the expert system prototype development process require corresponding alterations to the website design. Fourthly, the expert system interfaces, databases, and web pages are implemented. During the expert system’s validation and verification procedure, human experts and users who test the system may discover potential issues with the system.

### 2.2. Information Management System

**Figure 2:**
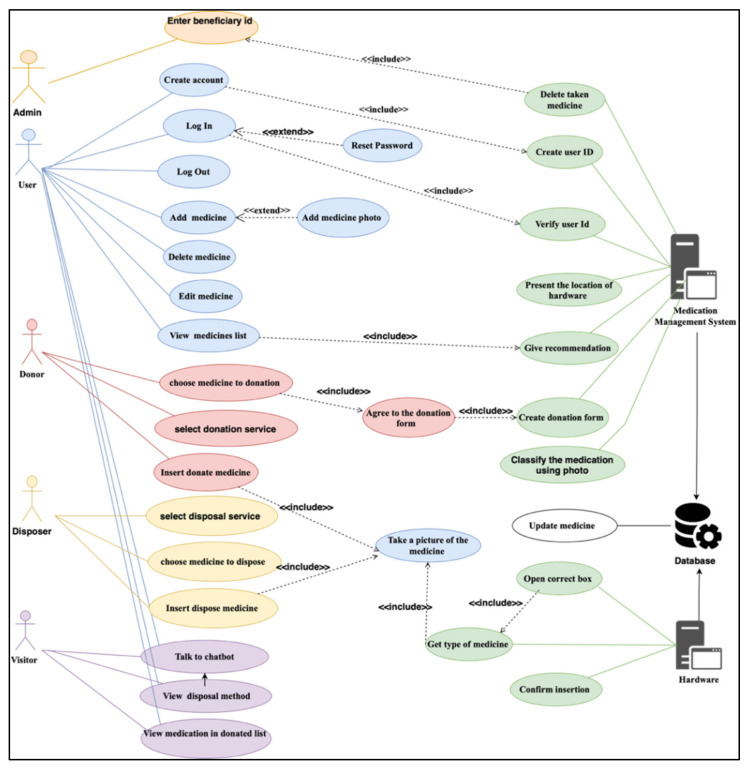
The user case of the medication management system.

The database was developed using the SQL server to store the medication information that is used by the inference engine. The database also stores medication data added by each user as shown in Figure 2. The system design and database are described in Supplementary Section S2. It includes the database and sequential design of the donation and disposal process. 

### 2.3. Chatbot

A chatbot is a computer program that interacts with people and simulates and processes human conversation (whether written or spoken), allowing people to interact with digital devices as if they were talking to a real individual. Chatbots can be as simple as a project that answers a basic query with a one-line interaction, or as complex as digital assistants who learn and evolve to import higher levels of personalization as they collect and process data.

The chatbot gives the user choices to choose from. The main menu contains four choices which are: (i) important information, (ii) knowing the validity of the medication, (iii) medication disposal method, or (iv) rephrasing user questions. Firstly, the important information contains details about how to dispose of medications using the hardware device, how to donate through the device, and how to take medications from the donated list. If the user chooses one of them, the chatbot will give all the information that the user needs. Secondly, knowing the validity of the medication developed based on the knowledge collected from the documentation, the chatbot asks the user some questions about the medication, then based on the user’s answers the chatbot will tell the user the validity of his/her medication. The knowledge is used from the flowchart. For example, the chatbot would ask the user to select the type of his/her medicine and the four types of medications were listed: “pills, drops, syrup, or cream”. If the user selected pills, the catboat would ask whether the pills were stored in packed blisters. Based on the user’s answer, the recommendation should be displayed, such as “great, the medication is useable until the expiration date”. Thirdly, the medication disposal method contains important information about how to dispose of the expired medication correctly based on the type of medication. Finally, the last choice is rephrased as “user question”. If the user cannot find his/her question in the choice, he/she can repeat his question to the chatbot and receive an answer. All multiple choices, questions, and answers are contained in Supplementary Section S3.

### 2.4. Classification Model

#### 2.4.1. Dataset

The 239 samples were taken from the Registered Human Medicines List dataset, which can be accessed from the open data resource [17] (see Supplementary Section S4). The medication images from different sides were collected and added to the dataset. All medications are registered and contain the trade name, medicine type, package type, storage conditions, manufacturer name, and image of medicines. All images were classified into four categories: drop, pills, syrup, and cream.

#### 2.4.2. Sequential Model Process for Image Classification

Keras is an open-source neural network library written in Python. Keras allows us to easily build, train, evaluate, and execute all sorts of neural networks. The core data structure of Keras is a model, which lets the user organize and design layers. Sequential and functional are two ways to build Keras models. The sequential model is the simplest type of model, a linear stack of layers. Firstly, download and explore the dataset that the model is used for training and testing and preprocess the dataset. Then, the data is divided into training 80% and 20% for testing. Secondly, configure the dataset for performance and make sure to use buffered prefetching that could yield data from disk without having I/O become blocking. Thirdly, standardize the data. The RGB channel values are in the (0, 255) range. This is not ideal for a neural network; to make the input values small, the standardized values should be in the (0, 1) range by using a rescaling layer, and resize images using the image size argument of image dataset from a directory or include the resizing logic in the model using the resizing layer. Fourthly, create the model, compile it, and train it using the dataset. If there are differences in accuracy between training and testing, the accuracy is noticeable, which is a sign of overfitting. When there are a small number of training examples, the model sometimes learns from noise or unwanted details from the training examples; to an extent that it negatively impacts the performance of the model on new examples. Data augmentation takes the approach of generating additional training data from our existing examples by augmenting them using random transformations that yield believable-looking images. This helps expose the model to more aspects of the data and generalize better.

### 2.5. Prototype

Software flowcharts (see Supplementary Section S5) illustrate how the website works. It is divided into two types of users: The first type is the user who visits the site and has only some authority, including: viewing the list of donated medicines and knowing the places in which they are available through the map and writing inquiries in the chatbot. The visitor could learn how to properly dispose of the medicine depending on the type. The second type of user who has a membership on the site and has more authority, namely: viewing the list of donated medicines and displaying the places of available medication through the map. Modifying drug data and removing or adding a new drug to the medication list. Writing inquiries on the chatbot program. Learning how to properly dispose of the medicine depending on the type.

The hardware was designed with the Arduino Integrated Development Environment (IDE) (Arduino, Ivrea, Italy). This was used for creating the simulation and is a cross-platform application (for Windows, macOS, Linux). TinkerCad was also used, which is an online collection of software tools from Autodesk (San Rafael, CA, USA) that enables the creation of 3D models. The hardware screens were linked to the website; the user should be registered to log in to the device services.

## 3. Results

Medication information was collected to create a knowledge base for the proper management of medication. The knowledge is demonstrated in flowcharts. The form of if-then rules has also been used to represent the knowledge. The web-based expert system has been developed to automate the recommendation and educate consumers about medication disposal. Using the website for managing the medications either disposing or donating. The chatbot was constructed to interact with consumers using the knowledge base. Finally, the prototype device was implemented to automatically verify and classify medications based on their type and validity using artificial intelligence methods. The system has two main components: software and hardware. The software includes a web-based expert system and chatbot. The hardware contains a classification model.

### 3.1. Web-Based Expert System User Interface

**Figure 3:**
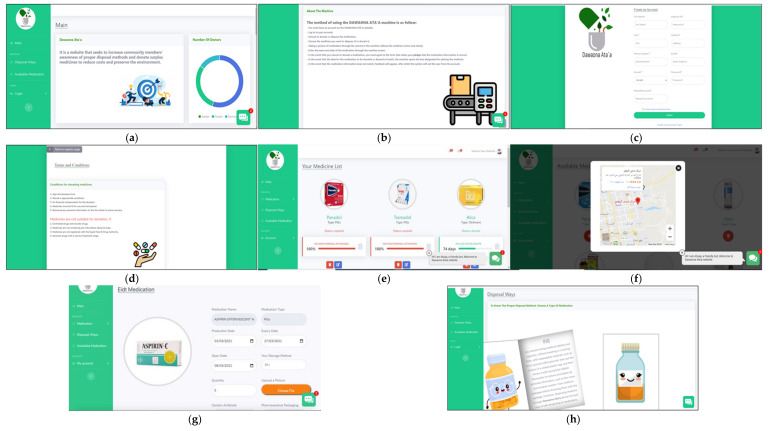
(**a**) Main page; (**b**) information about device; (**c**) registration page; (**d**) donation rules; (**e**) user medications list page; (**f**) location of machines containing the medicine; (**g**) edit the medication; and (**h**) instructions for disposal page.

For designing the web-based expert system, the HTML (Hypertext Markup Language) (WHATWG, Washington, DC, USA) and CSS (Cascading Style Sheets), Hypertext Preprocessor (PHP) (Zend Technologies, Toronto, Canada) and JavaScript (ECMAScript, United State), Bootstrap (Bootstrap Core Team) and Hostinger (Kaunas, Lithuania) were used. For the database, XAMPP (Apache, United State) is an abbreviation for cross-platform, Apache, MySQL, PHP, and Perl, and it allows us to build a website on a local web server on a computer. PhpMyAdmin (Maguma) was also used, which is a free software tool written in PHP that is designed to manage MySQL over the Internet. The web-based expert system functional and non-functional requirements are listed in Supplementary Section S6. The web-based expert system also had three types of users: admin, members, and visitors. Admin, member, and visitor are all important parts of this study and the medication take-back program. Participants in each of these activities must meet several criteria before being allowed to participate. Admin can be a pharmacy, a hospital a private clinic. The admin also had the authority to deliver the donated medicine and had only one page: give medication, he/she also could access all member’s accounts. The member had log in/out, main, disposal way, available medication, chatbot, medication list pages. The visitor shared the member in main, disposal way, available medication, and chatbot pages. The website is named “Dawaona Ata’a” which means medication givers. Some of the website pages are demonstrated in Figure 3.

**Figure 4:**
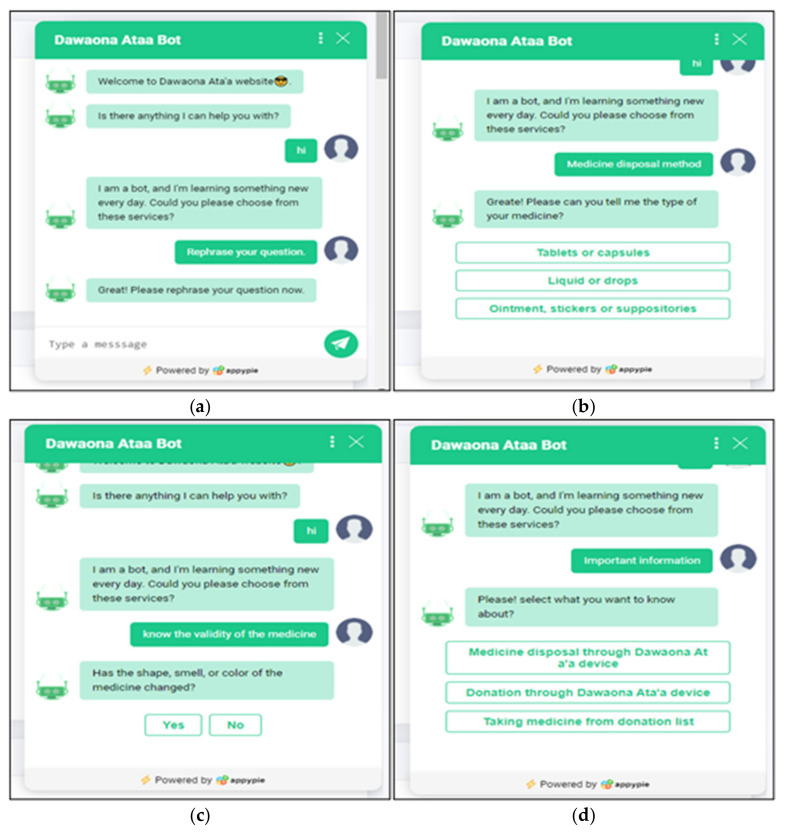
(**a**) Rephrase question: this option allows the user to rewrite the question again; (**b**) medicine disposal method: this option allows the user to choose the type of drug they want to know how to get rid of; (**c**) know the validity of the medicine: this option shows the user the validity of this drug based on some questions that the chatbot will ask the user, for example, has the drug changed color?; (**d**) important information: the user could select one out of three options.

Appy Pie^TM^ App Maker (Abhinav Girdhar) tool was used to create a chatbot and develop possible questions from users and answer multiple-choice questions which were developed based on disposal, donation, and validity of the medicine and some other information. Examples of the screenshot are shown in Figure 4.

**Figure 5:**
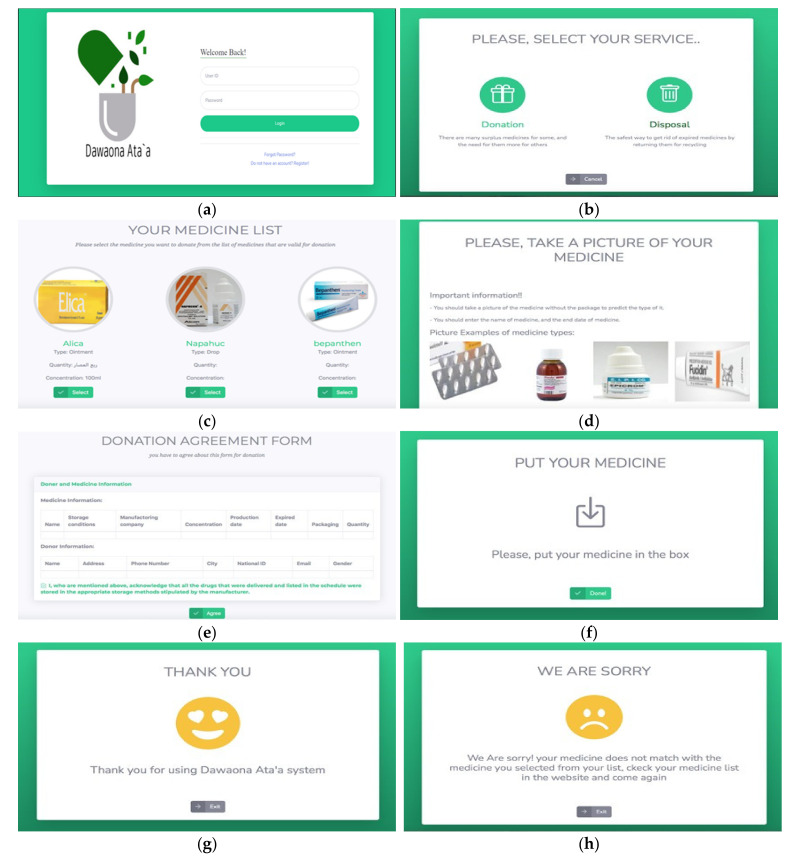
(**a**) Login page; (**b**) service page; (**c**) medication list; (**d**) guideline to take a picture; (**e**) donation agreement form; (**f**) insert the medication; (**g**) positive and (**h**) negative feedback.

In the hardware, the non-registered users of the site cannot use this device because their data is not available in the database. The users can log in through the iPad with hardware, and then two options will appear, which are donating a drug or disposal of a drug. If the user chooses to donate, his/her list of medicines will appear on the screen. Then, the user selects the medicine that they want to donate. The donation form is displayed on the screen and must be filled out and agreed with policies. The machine also asks the user to place the medicine in front of the camera for verification. On this page, the classification model will be loaded and after the send button is clicked, the system will pass the image through the model to obtain a prediction about the type of medicine. After obtaining the type from the model, and the name and expired date of the medicine from the user, the system compares it with the information from the database that the user selected on the website (see Figure 5). After verification, the system will open the correct box for the donor based on the type of medicine or the garbage box for the disposer. If there is any error in the entered data from the user, the system will show negative feedback to the user. If the user does not fill in the information or does not capture the medicine, the system will give error messages.

### 3.2. Implementation of the Sequential Model Process for Image Classification

**Figure 6:**
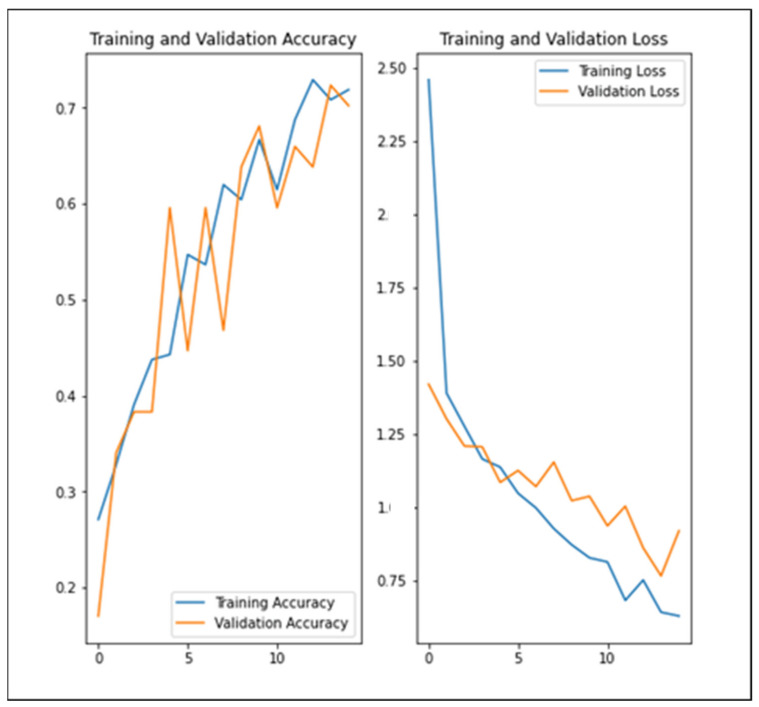
Visualize the training results.

The classification model was built using Python. There were four categories according to its type (pills, syrup, cream, or drops). This property is used while using the device for donation or disposal services. When the user captures the medicine in the device, the system takes the image and passes it through the classification model to predict the type of this medicine. Then, compare it and retrieve additional information about the medication from the database. The result of the prediction is one of the medication types and this is used for opening the correct box of medicine in the device. After applying data augmentation and dropout techniques, there is less overfitting than before, the training and validation accuracy are closely aligned, and the model has achieved around 70% accuracy on both validation and training sets as shown in Figure 6.

### 3.3. Hardware Simulation

**Figure 7:**
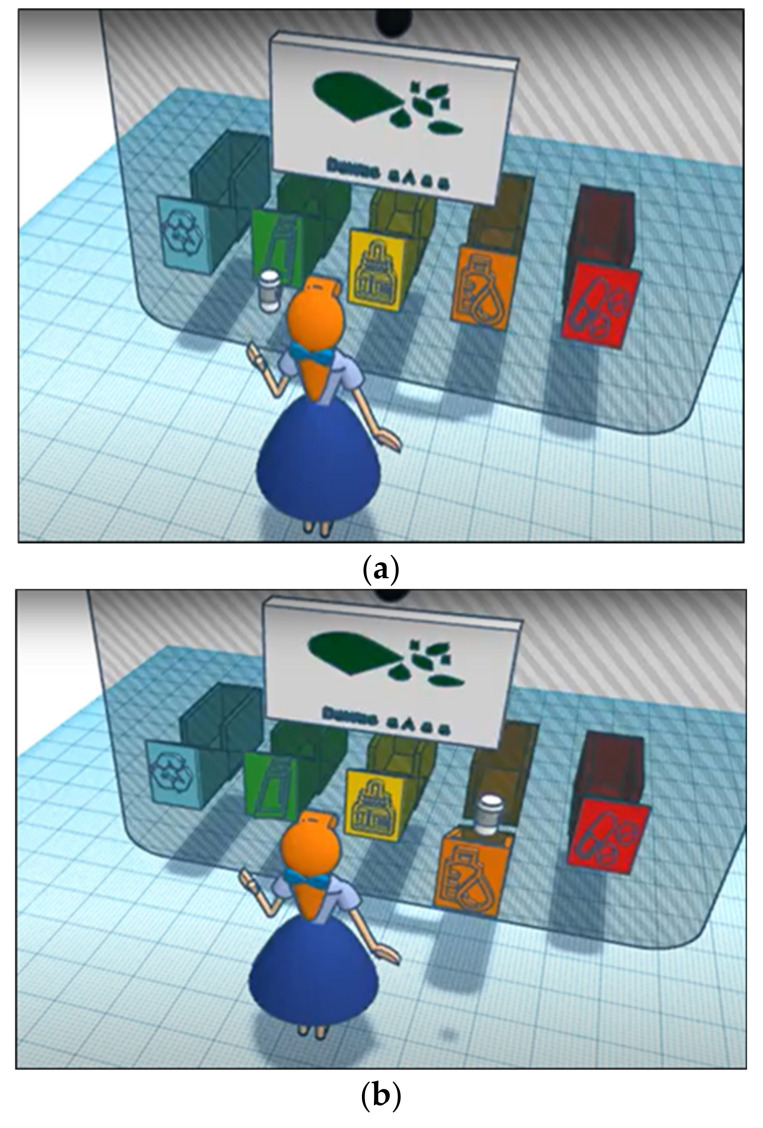
(**a**) The 3D model takes a picture of medication and (**b**) the 3D model opens the correct box.

An Arduino simulator is used to design the device which contains: an Arduino UNO (Arduino, Ivrea, Italy) connected to a Wi-Fi shield connected to an (Apple, CA, USA) to receive commands from it. It also contains five servos attached to boxes for the four types of medicines (pills, syrup, drops, or cream) and a fifth box to remove expired medicines. The 3D simulation is demonstrated in Figure 7. 

The iPad will display the screen to log in to the account, the classification model is integrated into the website and will be called when the user completes all required forms and place the medication in front of the camera.

## 4. Discussion

In recent years, the use of pharmaceutical products has increased significantly worldwide. The increasing use and improper disposal of pharmaceutical products around the world have drawn attention to the negative impacts on the environment. Firstly, the Kingdom of Saudi Arabia expends billions to provide treatment while the waste of medications results in a massive loss of financial resources. Secondly, some people need treatment and medication but cannot afford them. Many people may not know of the best ways to dispose of unused and expired drugs and there is a need to make the public aware of this and to assist in the proper gathering of unused pharmaceutical products [5,18,19]. Bridging all these issues and addressing them through the deployment of the website and device was the focus of our research. This research considered the importance of awareness that includes an educational website regarding the methods of proper handling of medicines in relation to preservation and storage operations and safe ways to dispose of them and about the expiration date written on the medicine packages to encourage patients and their families once they stop using the medicines to bring them to the medication take-back point instead of disposing of them in unhealthy and unsafe ways for the environment and society.

The first strength is that the three types of users who followed the rules and information on the website were highly committed to organizing the medication life cycle. Starting to add the medication details and the status of the medication are changed once the medication had expired. The website educated the users about the proper disposal method based on the medication type. The other option was to denote the unused medication by linking the process of medication management. Using the website to store the medication details, and displaying the donated list on the website, putting the medication in the device after signing the agreement of the policies of donation. This regulates the process of collecting medications and giving them to the needy under healthcare control since the patient purchased the medication until he/she decided to dispose of or donate them.

The other advantage is preventing medicines from reaching streams and rivers or water sources when pouring them down the drain or toilet. When flushing medication down the bathroom sink, prescription and OTC medications can leak into the soil and groundwater. Although this research provided a safe and ecologically friendly method of disposing of unneeded pharmaceuticals, it is important to obtain the recommendation to use biodegradable chemicals and environmentally friendly packaging materials, which can be recycled or made from recycled materials, which could help in further reducing pharmaceutical pollution [20].

The weakness in this study could be reflected in consumer behavior. For example, users may have difficulty following instructions and proper procedures for medication disposal or donation. In addition, users might not have time to bring the medications to take-back devices. Although the website included all the information about the proper disposal methods, users might lack concern about the risks resulting from improper disposal methods and their impact on the environment.

Data loss and the accumulation of medicines in the devices are possible problems in this research. Data can be rendered unreadable by humans and software alike if it is infected with a virus or suffers physical harm. A direct result of losing the database is the time and money it takes to repair or recover data; in our device, this is critical. All medications should be disposed of since this problem might affect humans. The second problem is the accumulation of medicines in the devices. Even though the purpose of this study was to minimize the harmful impact of environmental contaminants, the study did not consider the solution to this problem. Thus, the admin should manage this by contacting healthcare-related industries. The medication must be properly managed in line with all applicable government policies. Smale et al. [20] suggested strategies to solve problems such as waste reduction, reuse, and recycling, which are all important components of a circular economy strategy, but they need not be the only ones employed.

## 5. Conclusions

This study proposed supporting the proper management and disposal of unused and expired medications by teaching people how to dispose of medicines and pharmacies by using a suitable machine for the proper disposal and donation of medications. The web-based expert system and chatbot provided education for consumers regarding correct medication disposal practices and contributed to increasing awareness of the possibility of donating usable medicines. The prototype device was designed for collecting medication to automatically classify medications for proper disposal and donation.

The study included two parts (software and hardware): (i) the web-based expert system containing a user medicines list, medication disposal/donation information, and a chatbot. The user could display information about the medicine validity, edit the medicine list, and dispose or donate of medicine. The user could also inquire about the appropriate way to dispose of the medication based on its type (drops, ointment, liquid, tablets) and validity (expired or usable). The user could display the list of donated drugs and saw the location of the devices (hardware); (ii) the prototype device was used to collect and dispose of medicines after verification. This device used a classification model and image for classification verification. The initial prototype design of the web-based expert system would reduce the risk of improper disposal by providing substantial guidance on the safe disposal of unused medicines. The donation was also supported by using an organized method of collecting unused medicines. A limitation of the web-based expert system is the manual entering of medication information which takes a long time, is tedious and repetitive, and increases errors; for example, failure to enter the correct information about medicines and their validity with credibility, such as the incorrect storage method. In the future, the system can be improved by detecting the name and date of the drug from the image and reducing the number of manual inputs by the user. The classification model can also be improved by evolving the model automatically to include more medicines in the dataset with all upcoming medications that can be added by the users. In addition, both the website and the device can be enhanced to support other languages.

## Figures and Tables

**Table 1:** The common medication types and their descriptions.

Medication Type	Descriptions
Liquid	The active component of the medication is mixed with a liquid to make it easier to handle or better absorb. A liquid can also be referred to as ‘mixture’, ‘solution’, or ‘syrup’.
Tablet or pills	The active ingredient is mixed with another material and pressed into a solid round or oval form.
Capsules	The active ingredient of the medicine is stored within a plastic shell that slowly breaks down in the stomach. Some need to be swallowed whole medicine because is not absorbed until the stomach acid breaks down the capsule shell.
Topical medicines	Topical drugs that are creams, lotions, or ointments applied directly to the skin. The active component of the medicine is combined with another material, making it easier to apply to the skin. They come in different types such as bottles or tubes depending on the form of medicine.
Drops	These are usually used where the active component of the medicine works better if it enters the infected region directly. They prefer to be used for eyes, ears, or noses.

## Data Availability

Publicly available datasets were analyzed in this study. This data can be found here: https://data.gov.sa/Data/mn_MN/dataset/registered-human-medicines-list/resource/0c57e5d6-e209-49bf-b1a1-310a6861432f accessed on 2 March 2021.

## Supplementary Materials

The following are available online at https://www.mdpi.com/article/10.3390/ijerph19052875/s1, Section S1. Knowledge Acquisition; Section S2. System Design; Section S3. Multiple choice questions and answers; Section S4. Dataset; Section S5. Prototype; Section S6. Requirements.
