# Supplementary material for: An Intelligent System for Proper Management and Disposal of Unused and Expired Medications
Source: Int J Environ Res Public Health. 2022 Mar 1;19(5):2875. doi: 10.3390/ijerph19052875 (PMC8910225; doi:10.3390/ijerph19052875)
Supplement: Supplementary file 1 [file ijerph-19-02875-s001.zip › ijerph-1522162-supplementary.pdf]

Supplementry Document

An Intelligent System for Proper Management and Disposal of Unused and Expired Medications

Haneen Banjar, Rahaf Alrowithi, Sara Alhadrami, Esraa Magrabi, Reema Munshi, and Mayda Alrige

S1. Knowledge Acquisition

The Saudi food and drug authority [1] has published a document to present donation forms and procedures. The documentation included forms which are used to organize the donation process such as figure 1 which represented medicines donation form in Arabic. In addition, figure 2 shows medicines receipt form for beneficiary form. The procedures for donation as the following:

1. The donor's signature should be clear on the donation form, which ensure that it is preserved and stored in appropriate conditions.
2. No financial compensation for the donation.
3. Medicines must be fit for use and not expired.
4. Removing any personal information on the the sticker to assure privacy.
5. The patient who takes medication must sign the medicines receipt form. It requests full details about the drugs to be considered for reuse.

Medicines are not suitable for donation, if:

1. Controlled drugs and narcotic drugs.
2. Medicines are not containing any information about its type.
3. Medicines are not registered with the Saudi Food & Drug Authority.
4. Sensitive drugs with a narrow treatment range.

مرفق رقم ١

(نموذج التبرع بالأدوية)

اسم المستحضر (التجاري والعلمي)	التركيز	الشكل الصيدلاني	الكمية	ظروف التخزين	تاريخ الانتاج	تاريخ الصلاحية	رقم التشغيلة	الشركة الصانعة

أقر أنا المدونة بياناتي أدناه بأن جميع الأدوية التي تم تسليمها والمذكورة في الجدول تخزينها بطرق التخزين المناسبة التي نصت عليها الشركة الصانعة.

التاريخ:

معلومات المتبرع أو الجهة المتبرعة:

الاسم: العنوان:

رقم الهوية (في حالة الافراد): معلومات التواصل:

التوقيع:

Figure 1: Medicines donation form in Arabic

مرفق رقم ٢

(نموذج استلام الأدوية للمستفيد)

اسم المستحضر (التجاري والعلمي)	التركيز	الشكل الصيدلاني	الكمية	ظروف التخزين	تاريخ الانتاج	تاريخ الصلاحية	رقم التشغيلة	الشركة الصانعة

أقر أنا المدونة بياناتي أدناه بعلمي التام بأن الادوية التي تم استلامها والمذكورة في الجدول هي أدوية رجيعة وأنني أتحمل كامل المسؤولية تجاهها و سأقوم بالتبليغ لدى الهيئة العامة للغذاء والدواء عن أية أعراض جانبية أو مضاعفات نتيجة استخدام هذه الأدوية.

التاريخ:

معلومات المستفيد أو الجهة المستلمة:

الاسم: العنوان:

رقم الهوية: معلومات التواصل:

التوقيع:

الصيدلي:

الاسم: التوقيع:

Figure 2: Medicines receipt form for beneficiary in Arabic

S2. System Design

The figure 4 included description and showed the implemented database:

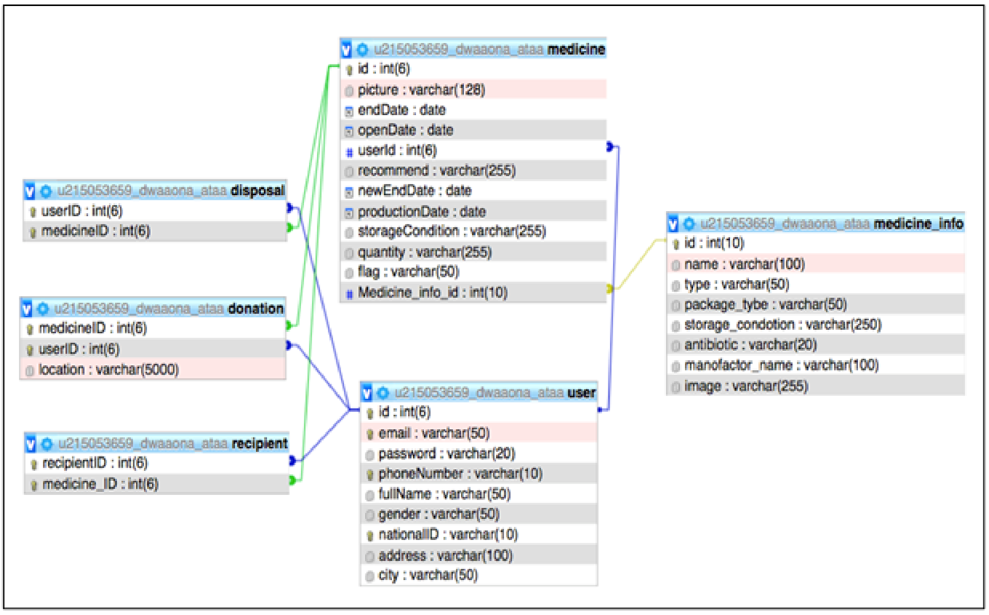

Figure 3: Database

Donation sequence diagram

Figure 4 shows the sequential for donation process. First, the doner should log in to the system by entering his/her ID and password on GUI, then the system will check the doner account in the database, if it does not find the system will display an error message. Second, after doner login, the system will display the main page that contains a donations and disposal services. After the donation was chosen, the system will get the doner medicine list from the database and display them on GUI. The doner selects the medicine that he wants to donate, the system will display a camera page. The doner should capture his/her medicine and enter the name and expired date. After that, the system will check of medicine by passing the picture through classification model to get the type, and compare the type, name, and expired date from doner with the medicine information from DB, if it is the same medicine that the doner chose from his/her list, if not the system will display an error message. Finally, the system will get doner and medicine information from the database, then create and display the

donation form to be agreed upon. After form agreed by the doner, the system will open the appropriate box for the medicine according to its type. After the doner puts the medicine inside the machine, the system will delete medicine from doner list, and the system will display thank you message then exit.

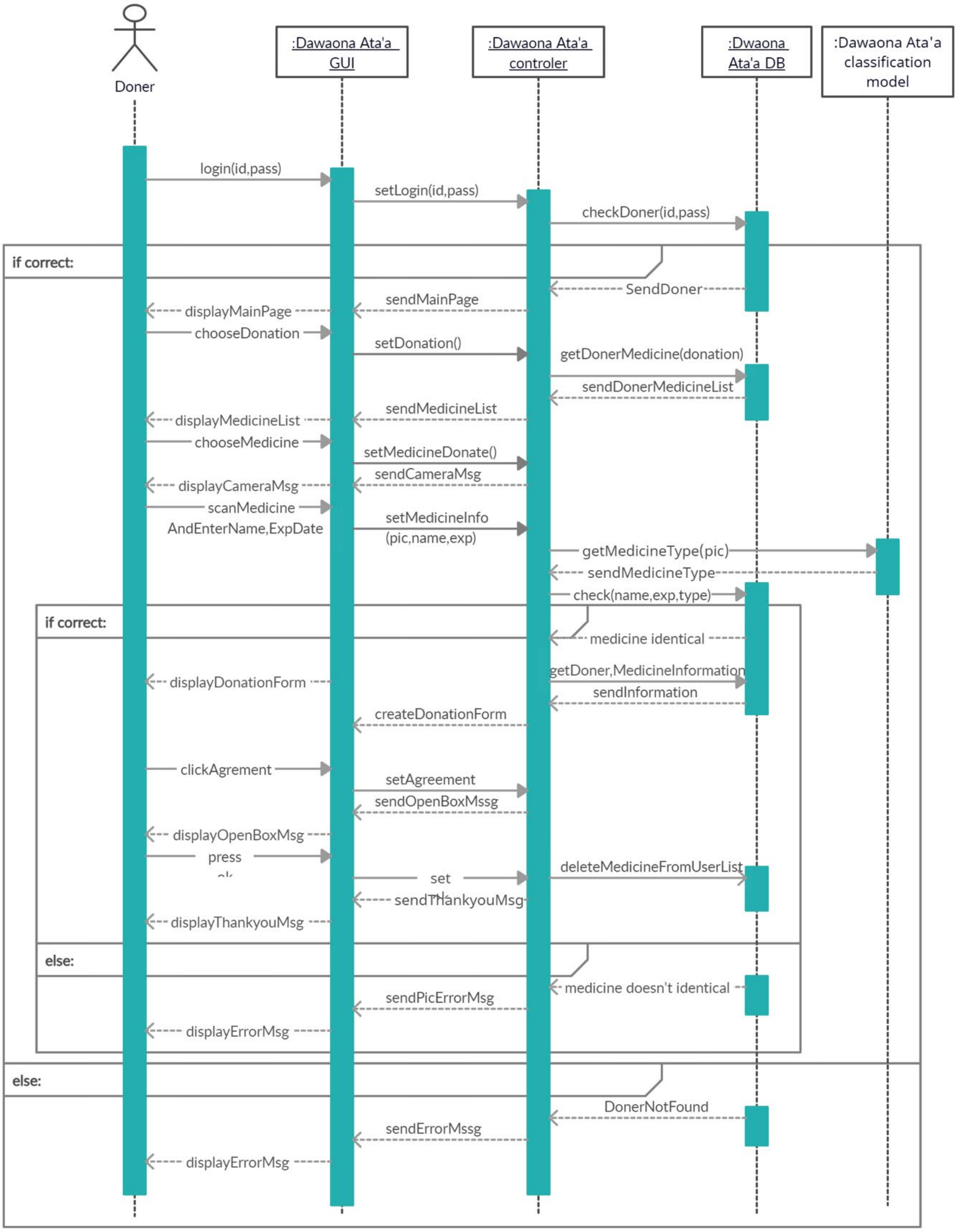

Figure 4:sequential design for the donation process

Medicine Disposal Sequence Diagram

Figure 5 shows the sequential for disposal process. First, the disposer should log in to the system by entering his/her ID and password on GUI, then the system will check the disposer account

in the database, if it does not find the system will display an error message. Second, after disposer login, the system will display the main page that contains a donations and disposal services. After the disposal was chosen, the system will get the disposer medicine list from the database and display them on GUI. The disposer selects the medicine that he wants to dispose, the system will display a camera page. The disposer should capture his/her medicine and enter the name and expired date. After that, the system will check of medicine by passing the picture through classification model to get the type, and compare the type, name, and expired date from disposer with the medicine information from DB, if it is the same medicine that the disposer choose from his/her list, if not the system will display an error message. Finally, the system will open the appropriate box for disposal medicines. After the disposer puts the medicine inside the machine, the system will delete medicine from disposer list, and the system will display thank you message then exit.

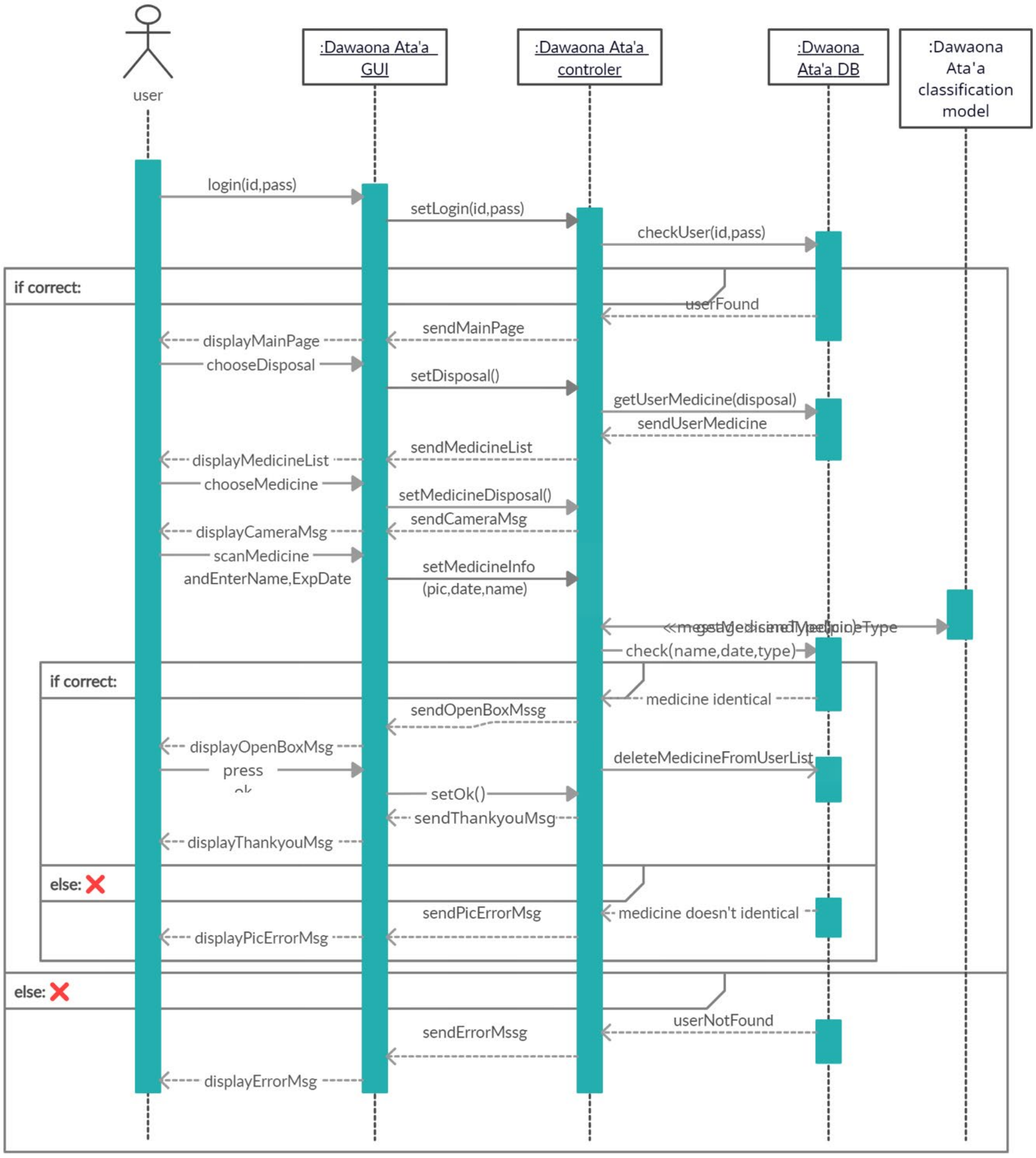

Figure 5:sequential design for the disposal process

S3. Multiple choice questions and answers

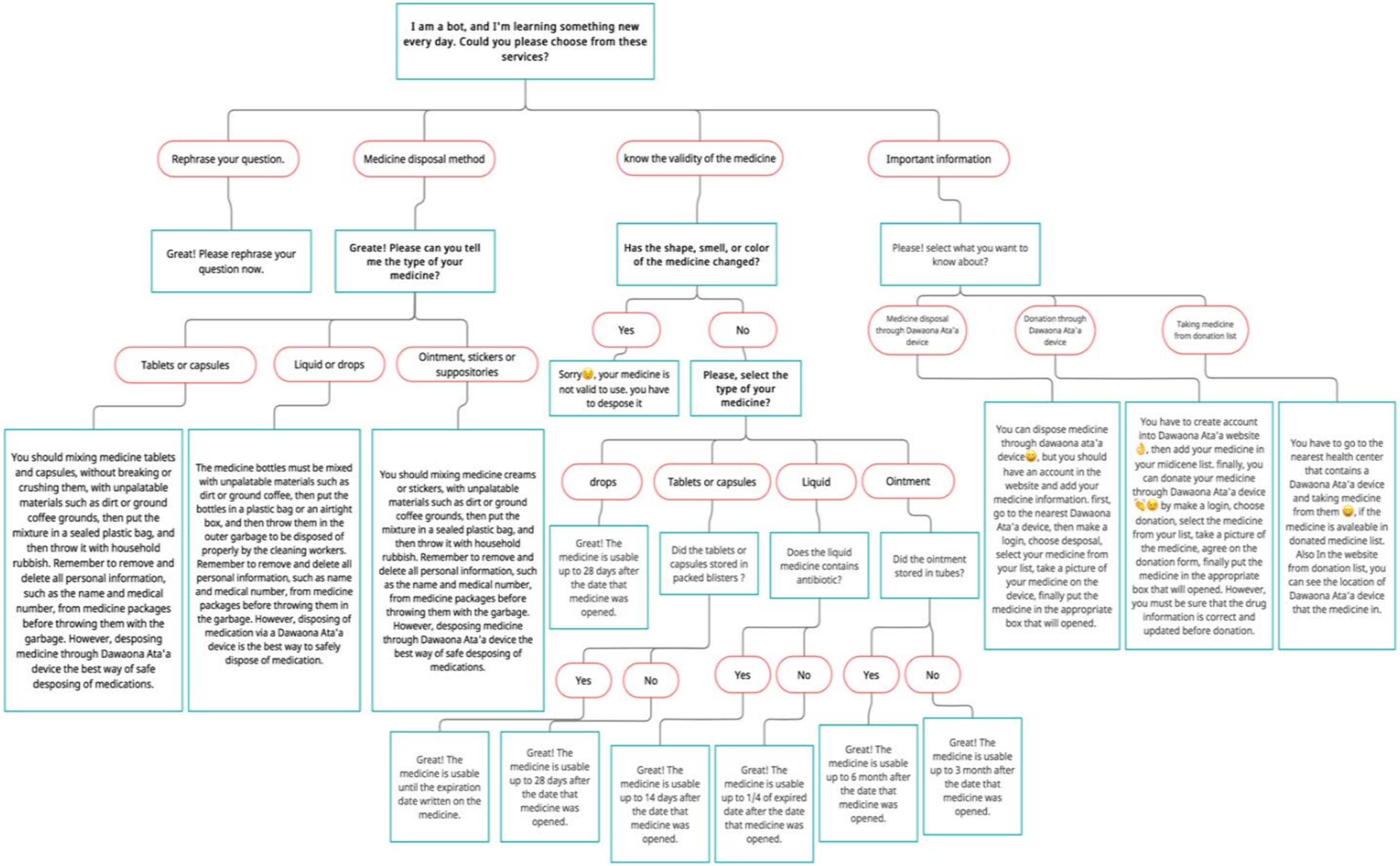

S4. Dataset

The 239 sample is taken from the Registered Human Medicines List dataset, which can be accessed from the open data resource [2]. The data set as presented in figure 7 included: dosage form, route of administration, package type, legal status, storage condition, image from right side, left side and front side.

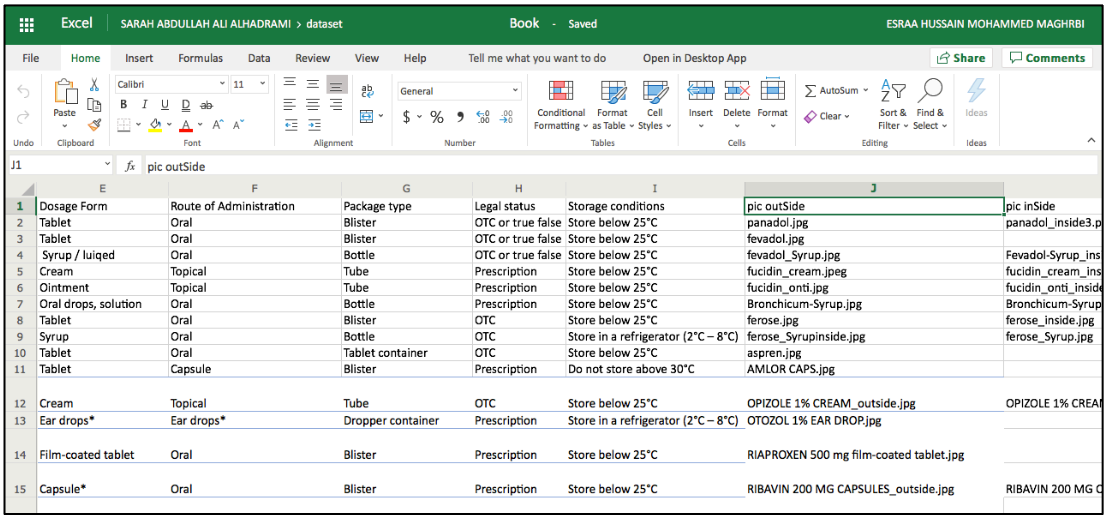

	E	F	G	H	I	J	
1	Dosage Form	Route of Administration	Package type	Legal status	Storage conditions	pic outSide	pic inSide
2	Tablet	Oral	Blister	OTC or true false	Store below 25°C	panadol.jpg	panadol_inside3.p
3	Tablet	Oral	Blister	OTC or true false	Store below 25°C	fevadol.jpg	
4	Syrup / luiqed	Oral	Bottle	OTC or true false	Store below 25°C	fevadol_Syrup.jpg	Fevadol-Syrup_ins
5	Cream	Topical	Tube	Prescription	Store below 25°C	fucidin_cream.jpeg	fucidin_cream_ins
6	Ointment	Topical	Tube	Prescription	Store below 25°C	fucidin_onti.jpg	fucidin_onti_inside
7	Oral drops, solution	Oral	Bottle	Prescription	Store below 25°C	Bronchicum-Syrup.jpg	Bronchicum-Syrup
8	Tablet	Oral	Blister	OTC	Store below 25°C	ferose.jpg	ferose_inside.jpg
9	Syrup	Oral	Bottle	OTC	Store in a refrigerator (2°C – 8°C)	ferose_Syrupinside.jpg	ferose_Syrup.jpg
10	Tablet	Oral	Tablet container	OTC	Store below 25°C	aspren.jpg	
11	Tablet	Capsule	Blister	Prescription	Do not store above 30°C	AMLOR CAPS.jpg	
12	Cream	Topical	Tube	OTC	Store below 25°C	OPIZOLE 1% CREAM_outside.jpg	OPIZOLE 1% CREAI
13	Ear drops*	Ear drops*	Dropper container	Prescription	Store in a refrigerator (2°C – 8°C)	OTOZOL 1% EAR DROP.jpg	
14	Film-coated tablet	Oral	Blister	Prescription	Store below 25°C	RIAPROXEN 500 mg film-coated tablet.jpg	
15	Capsule*	Oral	Blister	Prescription	Store below 25°C	RIBAVIN 200 MG CAPSULES_outside.jpg	RIBAVIN 200 MG C

S5. prototype

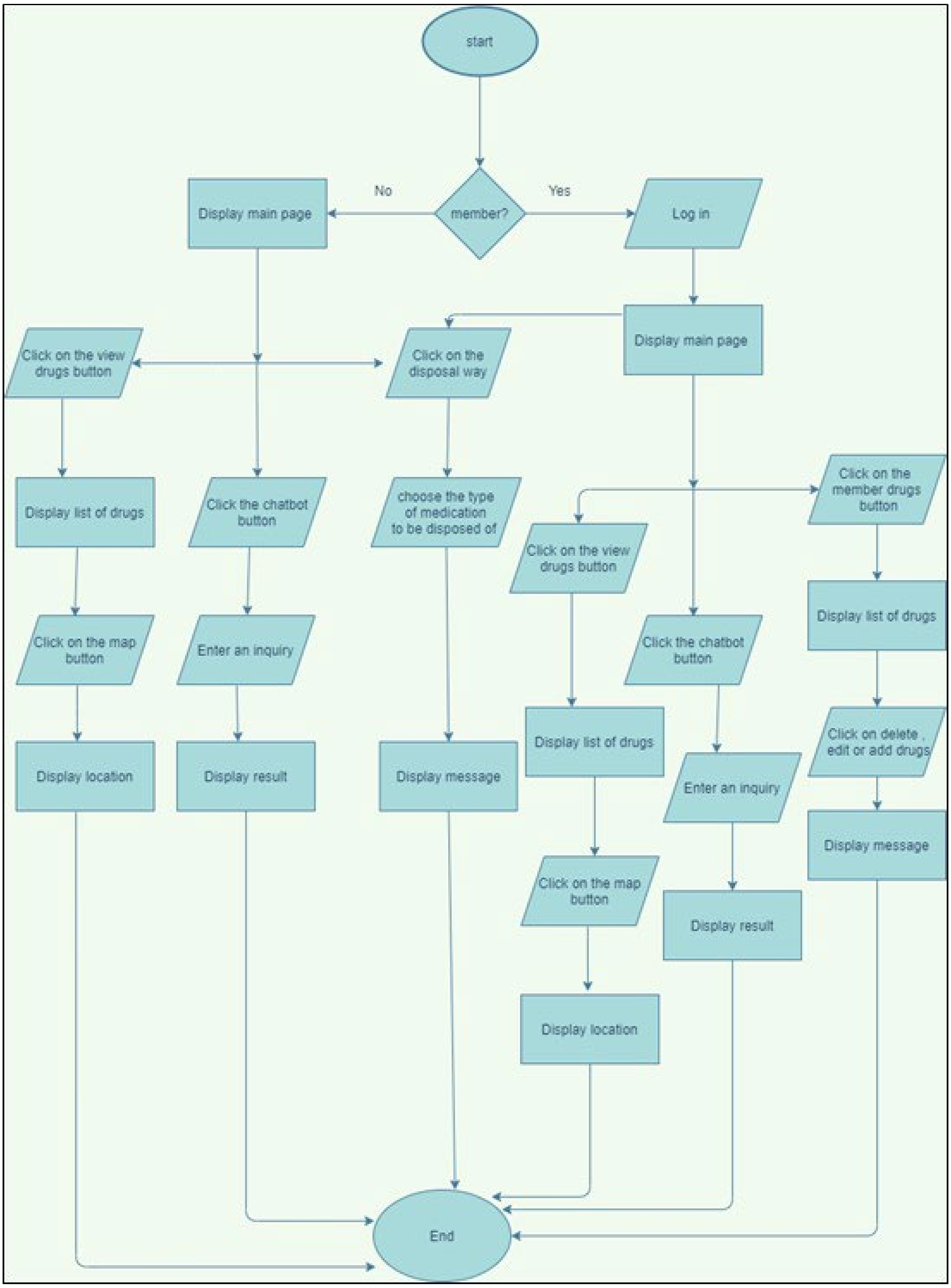

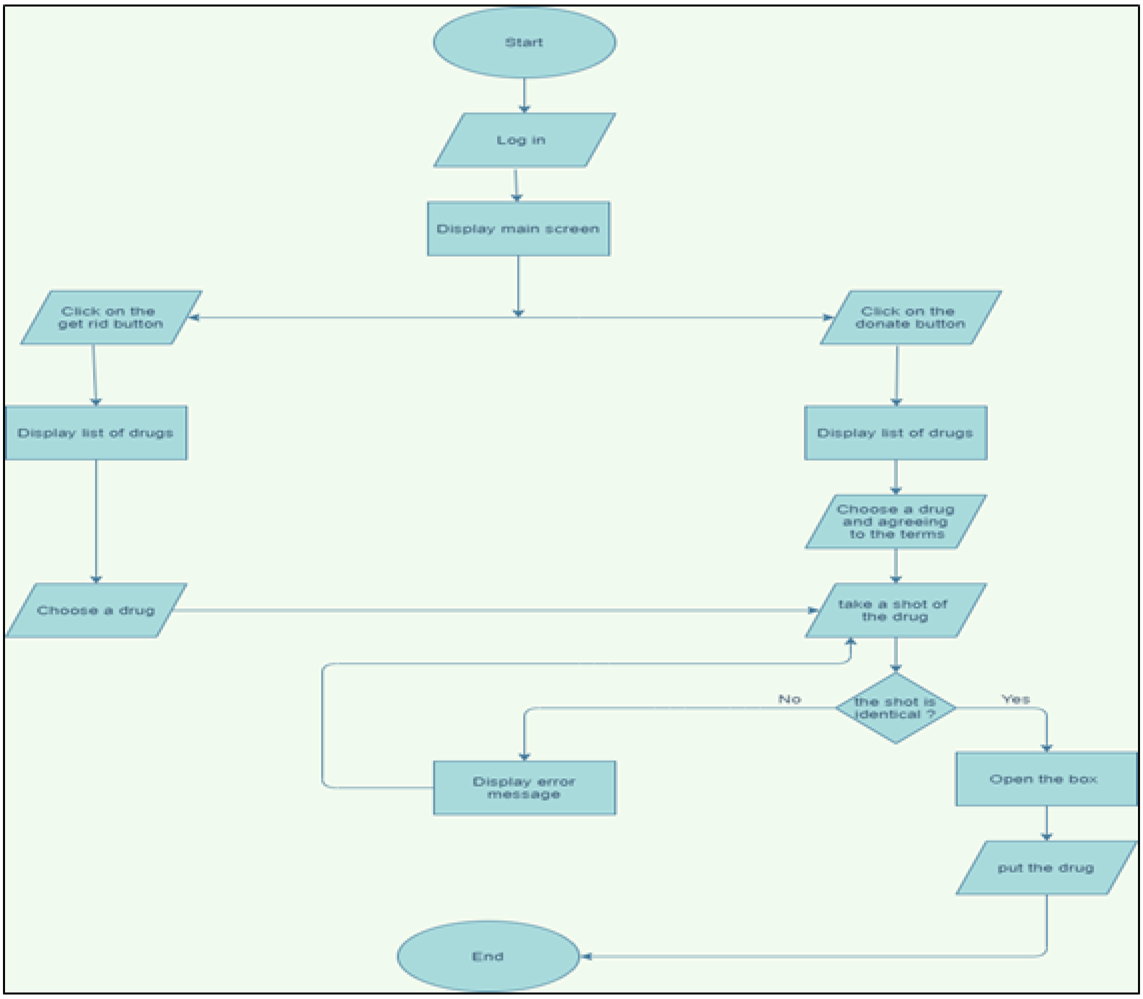

- **S6. Requirements**

Functional Requirements

- R1: The system shall allow the user to create an account.
- R2: The system shall create the user id and send it to the user email.
- R3: The system shall verify the user mobile number.
- R4: The system shall allow the user to login.
- R5: The system shall verify the user id when he/she logged in.
- R6: The system shall allow users to log out.
- R7: The system shall allow the user to reset his/her password.
- R8: The system shall allow the user to add/delete/edit medicines.

- R9: The system shall allow the user to add/ remove/edit medicine’s photograph.
- R10: The system shall allow users to view a list of medicines available to take for users.
- R11: The system shall allow users to view a list of his/her medicines.
- R12: The system shall allow user to search about disposal method of medicines.
- R13: The system shall allow the user to talk to the chatbot service to answer his/her questions and educate the user about the appropriate methods for expired medication disposal.
- R14: The system shall allow doner to choose medicine for donation from his/her medicine list that is suitable for donation.
- R15: The system shall allow disposer to choose medicine for disposal from his/her medicine list that expired or not suitable to use.
- R16: The system shall present the user's expired medicine list for disposal in ‘Dawaona Ata’a’ hardware screen.
- R17: The system shall present the user's medicine list that are suitable for donation in ‘Dawaona Ata’a’ hardware screen.
- R18: The system shall allow donor to fill out the donation form.
- R19: The system shall present the location of the nearest ‘Dawaona Ata’a’ hardware.
- R20: The system shall present the appropriate disposal methods for expired medicine to the user.
- R21: The system shall give a recommendation to the user whether his/her medication has expired, not suitable to use, almost done or good for donation.
- R22: The system shall update user medicine list when disposal and donation.
- R23: The system shall display donation agreement form to the donor for his/her agreement.
- R24: The system shall present feedback messages to the user.

- R25: The system shall verify medicine by comparing a picture on 'Dawaona Ata'a' hardware with the medication name.
- R26: The system shall open correct box to the user to put the medicine according to its type.
- R27: The system shall confirm medicine insertion into 'Dawaona Ata'a' hardware.
- R28: The system shall allow doner/ disposer to enter medicine into 'Dawaona Ata'a' hardware.
- R29: The system shall allow doner/ disposer to take a picture of the medicine by the camera in the 'Dawaona Ata'a' hardware.

Non-Functional Requirements

- Availability: The system should be available in Arabic and the chatbot should be available in English.
- Usability: The system interface should be user friendly and conforms to web-applications.
- Accessibility: The system improves the methodology of disposal of unused and expired medication.
- Security: The system must protect the user account by choosing strong password.

References

[1] Saudi Food & Drugs Authority, "The mechanism for donating medicines and returning medicines.," 2020. [Online]. Available: https://old.sfda.gov.sa/ar/drug/resources/DocLib2/d3152020aaaa1draft.pdf.

[2] "Registered Human Medicines List." https://www.data.gov.sa/Data/%0Aen/dataset/registered-human-medicines-list.. (accessed Oct. 02, 2020).
